# Supplementary material for: The Whole-Genome Sequencing and Probiotic Profiling of Lactobacillus reuteri Strain TPC32 Isolated from Tibetan Pig
Source: Nutrients. 2024 Jun 16;16(12):1900. doi: 10.3390/nu16121900 (PMC11206325; doi:10.3390/nu16121900)
Supplement: Supplementary file 1 [file nutrients-16-01900-s001.zip › nutrients-3033077-supplementary.pdf]

Table S1. General features of the L. reuteri TPC32 genomes.

	L. reuteri **TPC32**
**Chromosome size (bp)**	2 214 495
**GC content (%)**	38.81
**tRNA**	75
**rRNA**	21
**Genomic island**	8
**Prophage**	3
**CDS**	2212
**CRISPR**	0
